# Editorial: Trends and challenges in plant biomonitoring, bioremediation and biomining

**DOI:** 10.3389/fpls.2024.1486752

**Published:** 2024-09-20

**Authors:** Ruslan Kalendar, Erika Levei, Oana Cadar, Marin Senila

**Affiliations:** ^1^ Helsinki Institute of Life Science HiLIFE, Biocenter 3, University of Helsinki, Helsinki, Finland; ^2^ National Laboratory Astana, Nazarbayev University, Astana, Kazakhstan; ^3^ Research Institute for Analytical Instrumentation, National Institute of Research and Development for Optoelectronics (INOE2000), Cluj-Napoca, Romania

**Keywords:** plant biomonitoring, plant bioremediation, plant biomining, pollution, sustainable

## Introduction

Environmental pollution is an urgent global challenge. Plant biomonitoring of soil, water, and air pollution is cost-effective, sustainable, and easy to use (Ye et al., 2017). The use of hyperaccumulator plants in environmentally friendly bioremediation technologies is a promising direction for the recovery of functional environmental elements. Despite the many studies on metal uptake by plants and the effects of pollutants on plants, the number of ready-to-use technologies for bioremediation and biomining is limited (Gavrilescu, 2022). The mechanisms involved in pollutant uptake and translocation and their enhancement by different methods are poorly understood; therefore, further investigations are needed for efficient pollution monitoring. One possible approach that could meet all these criteria is the use of plants for pollution biomonitoring, as they act as pollution integrators over long periods of time (Oladoye et al., 2022). Polluted sites need to be cleaned and restored; if possible, the valuable elements should be recovered (Kumar et al., 2021). Different plant species are suitable for all these processes, but there are no ready-to-use methods or standardized approaches are available (Jaskulak et al., 2020).

This Research Topic aims to bring together different types of research that could shed light on recent developments in biomonitoring, bioremediation and biomining in order to fill the knowledge gap needed to upscale the results of promising laboratory or field studies and to identify the challenges and opportunities involved. This research theme aims to explore the use of different plant species for biomonitoring of environmental pollution, recovery of valuable elements from the environment and bioremediation of the environment.

## Advances in the use of plants in biomonitoring of environmental pollution

Reduced biodiversity, severe soil erosion, and low agricultural productivity limit rapid economic development in the karst mountain areas (Otero et al., 2024). The loquat (*Eriobotrya japonica* L.) has become an economically valuable tree species utilized in rocky desertification remediation due to its exceptional drought tolerance, broad adaptability, and high nutritional and economic value. In a study by Hu et al., loquat plants were observed to preserve homeostasis by regulating physiological responses *in vivo* in response to soil nutrient modifications. This suggests that loquat has good adaptability in karst desertification environments. The soil and litter nutrient contents exhibited variation with increasing restoration years, with the highest values occurring mainly in May and July. The C, N, and P contents of loquat leaves were highest in year 10. Maintaining homeostasis by regulating internal physiological responses in the face of soil nutrient changes suggests that loquat is well adapted to karst desertification environments. Therefore, future research should apply scientific management that takes into account soil moisture and nutrients in different seasons to ensure that the results of karst desertification control can play a role in rural regeneration. Drought stress typically impedes plant growth, which complicates the process of revegetating slopes. In ecological restoration projects, gentle slopes and herbaceous plants are the dominant features. Guo et al. investigated the impact of incorporating exogenous arbuscular mycorrhizal fungi into vegetation material on the drought tolerance of slope plants. Inoculation of vegetation concrete with arbuscular mycorrhizal fungi resulted in increased plant height, root length, and biomass under drought conditions. A single inoculation has been observed to enhance aboveground biomass growth, while a double inoculation has been shown to facilitate belowground biomass growth. In the initial phases of slope ecological restoration, an increase in aboveground biomass is more beneficial to the revegetation effect of slope. Nevertheless, plant roots play a pivotal role in mitigation soil erosion and improving slope stability in slope ecological restoration projects. In the context of slow slopes or herbaceous plant-based slope environmental restoring projects, single inoculation methods are recommended. Conversely, for steep slopes or shrub-based slope ecological restoration projects, double inoculation methods are advised. The sustainability of slopes is contingent upon their stability. Consequently, future studies should consider the effects of arbuscular mycorrhizal fungal inoculation on the variation of plant root structure and erosion resistance of root-soil complexes in the steadiness of slope ecological restoration projects.

## Mechanisms involved in and factors that influence the environment to plant transfer of pollutants

Changes in tree species composition are an important aspect of forest succession (Seidl and Turner, 2022). In recent decades, there have been notable shifts in the tree species composition in subtropical forests in China. Specifically, there has been a decline in the prevalence of coniferous trees and an increase in the abundance of deciduous trees. For a variety of reasons, the populations of pine and fir tree species in Zhejiang Province have been declining for some time, and this trend is expected to continue in the long term. For a variety of reasons, the pine and fir tree species in Zhejiang Province have been experiencing a gradual decline, and this trend is expected to persist over the long term. In a recent study, Ji et al. projected that a nonlinear differential equation system model is an appropriate tool for simulating and predicting the forest tree classes composition in the macroscopic region of subtropical China. The model enables prediction at various time scales, including short-, medium-, and long-term. Furthermore, limited model analysis allows for in-depth exploration of currently unclear trends and the stability of forest tree species composition. The application of the model provides a foundation for the development of sustainable forest management policies. In another study, Lin et al. employed the maximum entropy method to predict the suitable habitats for *Aralia elata* and *Eleutherococcus senticosus* and to analyze the dominant factors affecting their distribution. In light of forthcoming climatic and land-use changes, the overall trajectory of suitable habitat for both commercial forest trees is predicted to undergo a northward and then a southward migration. These findings can be used to develop strategies for the conservation of resources and the sustainable utilization of *E. senticosus* and *A. elata*. It is recommended that areas with stable and suitable habitats be identified for the *in situ* conservation and breeding of the two economically valuable forest trees.

## Development in the use of plants in biomining

Crop height is an important agricultural parameter closely related to yield, above-ground biomass, and lodging. Data from ultrasonic sensors are easy to process, and this type of sensor is characterized by its cost-effectiveness, ease of portable installation, and suitability for prolonged exposure in the field environment, especially when compared to Light Detection and Ranging and Unmanned Aerial System Imagery (Bowler et al., 2022). Canopy tallness plays an indispensable role in the field of crop growth, serving as a crucial dynamic indicator in the decision-making process pertaining to agricultural management. Compared to the techniques most commonly employed for the measurement of canopy height, ultrasonic sensors offer a relatively inexpensive solution that can be deployed in the field for extended periods, facilitating the rapid processing of data. Zheng et al. demonstrated that both observation angle and plant density exerted a significant influence on the outcomes of ultrasonic measurements, while the effects of other factors on measurement accuracy were found to be inconsequential. Furthermore, a two-input-factor calibration model was developed to estimate canopy height in different years using the normalized difference between vegetation index and ultrasonic measurements. The least squares method was used to create the model, and the exactness of the ultrasonic measurement was meaningly enhanced when the measured canopy height and the normalized difference vegetation index were integrated. The results successfully combine stable and affordable marketable sensors with ground-based agricultural machine platforms, enabling the efficient and non-destructive achievement of crop height information.

## Recovery of economically valuable elements from plants

As an essential component of plant cell walls, lignin provides mechanical support for plant growth, enhances water transport, and helps defend against pathogens (Liu et al., 2018). As the most plentiful natural aromatic-based renewable resource on Earth, its biosynthesis has consistently been a focal point of research and remains a topic of ongoing investigation. In the study by Yao et al., the p-coumaric alcohol analogue and the coniferyl alcohol analogue comprising an alkyne group at the ortho site were produced and used for lignification *in vivo* and *in vitro*. The integration of these new lignin monomers was monitored by fluorescence imaging technique. It was demonstrated that the two monolignol analogues can be combined into dehydrogenated polymers *in vitro* and into flax cell walls *in vivo*. The results demonstrated that the deposition of H- and G-type lignin exhibited distinct patterns contingent on the specific cultivation time and precursor concentration. At the subcellular level, the deposited lignin initially manifests in the cell corners and middle lamellae, subsequently extending to the cell walls. Furthermore, lignin was also identified in the bast fiber. These novel molecules were shown to facilitate the precise localization of lignin in the polymerization process.

## Challenges in developing easy-to-use, cost-effective bioremediation methods

Lichens are recognized as highly effective biological indicators of air pollution (Yang et al., 2023). They have been widely used to detect a variety of airborne contaminants that come from both indoor and outdoor sources. In a recent study, Abas et al. explored the potential of lichens as a tool for detecting environmental tobacco smoke in a building setting. Understanding the spatial spreading of nicotine in indoor environments has significant implications for public health. This study validates lichens’ effectiveness as biological markers of indoor air pollutants, specifically nicotine. The findings suggest that elevated nicotine concentrations in lichens after approximately two months of exposure are associated with reduced lichen vital rates. Investigations of nicotine detection using lichens may facilitate the development of innovative monitoring techniques for evaluating indoor air quality. The incorporation of lichen-based sensors into transportable monitoring instruments or passive sampling tools could offer cost-effective and non-invasive techniques for continuous monitoring of nicotine concentrations in buildings. To address the challenges of low recognition, localization efficiency, and poor accuracy in multi-stage tomato recognition in complex environments, Fu et al. proposed a method based on an improved YOLO (You Only Look Once) version 8 model (Ultralytics, 2024). This improved model demonstrates more efficient and accurate recognition of tomatoes at different stages, providing a valuable technical reference for intelligent tomato harvesting.

In summary, this Research Topic aims to bring together different research efforts to highlight recent progresses in biomonitoring, bioremediation and biomining, to address knowledge gaps, and to identify challenges and opportunities for up-scaling promising laboratory or field study results.
